# Translational repression of 5’ TOP mRNAs in chronically activated CD8+ T cells *in vitro*

**DOI:** 10.1080/15476286.2026.2715355

**Published:** 2026-08-06

**Authors:** Erica S. Pledger, Navya Ambavaram, Lucas Ferguson, Jamie H. D. Cate

**Affiliations:** a Department of Molecular and Cell Biology, University of California Berkeley, Berkeley, CA, USA; b Innovative Genomics Institute, University of California Berkeley, Berkeley, CA, USA; c Department of Chemistry, University of California Berkeley, Berkeley, CA, USA; d Molecular Biophysics and Integrated Bioimaging, Lawrence Berkeley National Laboratory, Berkeley, CA, USA

**Keywords:** T cell, exhaustion, chronic activation, TOP mRNAs, translation regulation, mTOR, ribosome profiling

## Abstract

T cell exhaustion is a dysfunctional state that arises during chronic infections and cancer, characterized by impaired effector functions and sustained expression of inhibitory receptors. While transcriptional, epigenetic, and metabolic rewiring have been well documented in exhausted T cells, a comprehensive understanding of how translation is regulated in this state remains incomplete. To address this gap, we performed ribosome profiling and RNA sequencing on *in vitro* chronically activated human CD8+ T cells to globally assess translational control during a model of T cell exhaustion. Our analyses reveal a marked repression of 5’ terminal oligopyrimidine (TOP) mRNAs during chronic activation. Unexpectedly, we demonstrate that this translational repression occurs despite evidence of elevated mTOR activity. These findings uncover a previously unknown layer of translational control in exhausted T cells.

## Introduction

CD8+ T cells, or cytotoxic T cells, play a critical part in immune surveillance by detecting and eliminating infected and malignant cells. Upon acute antigen stimulation, CD8+ T cells undergo rapid transcriptional, translational, and metabolic reprogramming to support clonal expansion and differentiation into effector cells [1–3]. While the majority of these differentiated effector T cells eventually undergo apoptosis during the contraction phase of an immune response, a small surviving fraction gives rise to long-lived memory T cells. By contrast, CD8+ T cells encountering chronic antigen stimulation – such as in persistent viral infections or cancer – will gradually enter a state of dysfunction known as T cell exhaustion. This state is defined by the progressive loss of T cell effector functions, sustained expression of inhibitory receptors, and a failure to clear persistent antigen [4]. Exhausted T cells fail to produce key cytokines, including IL-2, IFNγ, and TNFα, and upregulate inhibitory receptors that negatively regulate T cell activation, such as PD1, LAG3, and TIM3 [4]. There has been significant progress in revealing the mechanistic underpinnings of T cell exhaustion, notably from an epigenetic, transcriptional, and metabolic perspective [5–7]. However, an understanding of global changes in translation that occur during T cell exhaustion is largely lacking.

Protein synthesis is one of the most energy-intensive processes in a cell and is therefore subject to tight regulation. Translational control plays a critical role in CD8+ T cell function and fate decisions [8]. Naive T cells are poised for activation by maintaining a reservoir of idle ribosomes and repressed mRNAs that can be rapidly translated following antigen stimulation [3]. Within just 6 hours after activation, T cells increase their protein synthesis rate by fivefold to support the burst in proliferation and increase in cell size [3]. This dramatic surge is driven by changes in the activity and expression of eukaryotic initiation factors (eIFs) as well as a substantial increase in ribosome biogenesis [3,9–12]. For example, naive T cells express little to no eukaryotic initiation factor 4E (eIF4E), but antigen stimulation induces its rapid expression [10,11]. Protein synthesis remains high for several days until the peak of the effector response, at which point CD8+ T cells rapidly downregulate translation as they cease to divide [1].

A major regulator of translation is the serine/threonine kinase mTOR (mammalian target of rapamycin). mTOR regulates protein synthesis by phosphorylating translation inhibitors and ribosomal subunits [8]. While this pathway globally promotes cap-dependent translation, a specific class of transcripts − 5’ terminal oligopyrimidine (TOP) mRNAs – are particularly sensitive to mTOR activity [13]. TOP mRNAs contain a +1 C directly after the 5’ m^7^G cap followed by a series of 4 to 15 pyrimidines and mostly encode ribosomal proteins and other components of the translational machinery [13]. In T cells, mTOR is a critical regulator of T cell differentiation, yet its influence on global protein synthesis appears more limited: pharmacological inhibition of mTOR during T cell activation only reduced protein mass by 26% in murine CD8+ T cells [10]. Instead, mTOR signalling likely has a highly selective role in regulating the expression of TOP mRNAs. These transcripts are dynamically regulated during T cell differentiation – their translation is repressed in naive T cells, rapidly increased following antigen stimulation, and then strongly inhibited at the height of the effector response, right before the contraction phase [1,3,14].

The state of protein synthesis in exhausted T cells has only recently begun to be explored. Studies in mice demonstrated that T cells undergoing exhaustion display a continuous rise in translational activity [15,16]. Using ribosome profiling, Liu et al. found that hypertranslation of transcripts encoding oxidative phosphorylation genes contributed to mitochondrial dysfunction in exhausted T cells [15]. Likewise, Wang et al. used mass spectrometry to reveal that heightened protein synthesis disrupts proteostasis, leading to the accumulation of stress granules and protein aggregates [16]. These findings suggest that dysregulated translation is a key feature of exhausted T cells and underscore the need for further investigation into protein synthesis control during exhaustion.

Here, we chronically activated human CD8+ T cells *in vitro* to recapitulate the phenotypic signatures of T cell exhaustion. We then used ribosome profiling and RNA sequencing to probe their translational landscape. We observed a striking repression in the translation of TOP mRNAs following chronic activation. Unexpectedly, we also provide evidence that mTOR is more active in chronically activated T cells than in transiently activated T cells. Collectively, our results reveal that exhausted CD8^+^ T cells possess a unique translational programme, alongside the well-characterized epigenetic, transcriptional, and metabolic programmes.

## Results

### *In vitro* chronically activated CD8+ Tcells show hallmarks of Tcell exhaustion

**Figure 1.**
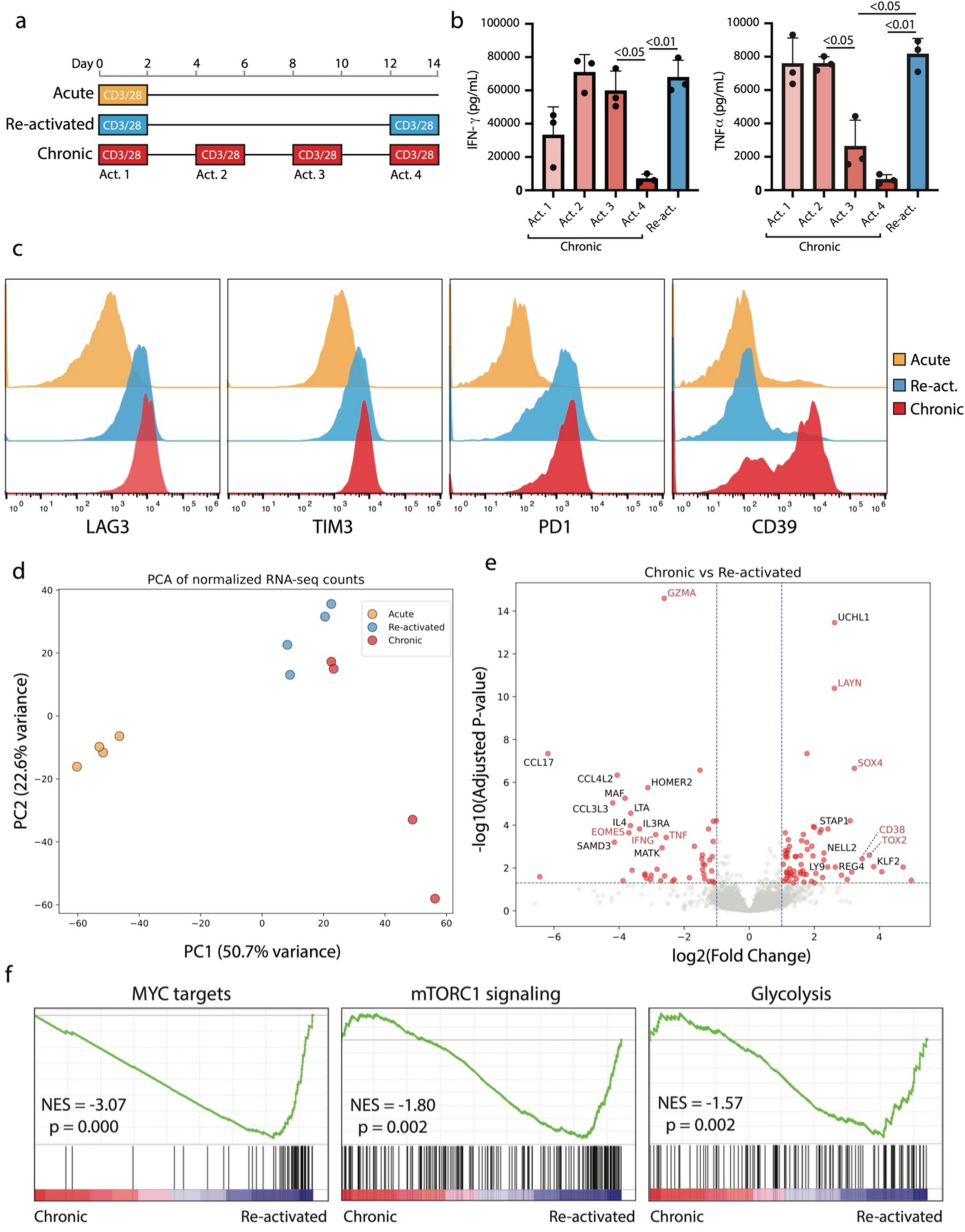
*In vitro* chronically activated CD8+ T cells show hallmarks of T cell exhaustion.

We first sought to establish a method for generating exhausted CD8+ T cells *in vitro*. We surveyed the literature for simple protocols that reproducibly produce large quantities of T cells that exhibit the molecular and phenotypic features of T cell exhaustion. An approach used by numerous groups is to chronically stimulate T cells with antibodies targeting the T cell receptor (TCR), bypassing the need for antigen stimulation [15–19]. Following this strategy, we purified CD8+ T cells from human peripheral blood mononuclear cells (PBMCs) of four healthy donors and activated them using anti-CD3 and anti-CD28 antibodies over the course of 2 weeks (Figure 1(a)). Our protocol employs a series of four 2-day activations separated by 2 days of rest, during which we remove the anti-CD3 and anti-CD28 antibodies to maintain cell viability and promote expansion (Figure 1(a), chronic). For comparison, we also activated CD8+ T cells for 2 days and either let them rest for the remaining 12 days or re-activated them on the last 2 days (Figure 1(a), acute and re-activated). These three conditions are herein referred to as T_chron_, T_re-act_, and T_acute_ cells.

(a) Schematic for *in vitro* generation of T_acute_, T_re-act_, and T_chron_ cells by anti-CD3 and anti-CD28 antibody activation. Each 2-day activation is indicated in T_chron_ (Act. 1, Act. 2, etc). (b) IFN-γ and TNFα secretion of T_chron_ and T_re-act_ measured by ELISA (*n *= 3 donors, error bars represent mean ± standard deviation). Of note, T_re-act_ (in blue) shows the final activation (ending on day 14), not the initial activation. *p* values were calculated using unpaired two-tailed *t* tests. (c) Representative flow cytometry histograms of the inhibitory receptors LAG3, TIM3, PD1, and CD39 (*n* = 4 donors). (d) Principal component analysis of RNA-seq data from T_acute_, T_re-act_, and T_chron_ cells in four donors. (e) Differential expression analysis of RNA-seq data comparing T_chron_ to T_re-act_ cells. Genes with significant changes in expression are in red (|log_2_(fold change)| >1 and adjusted *p*-value <0.05). (f) GSEA of MYC target genes, mTORC1 signalling pathway genes, and glycolysis genes in T_chron_ versus T_re-act_ cells.

Next, we sought to determine if our T_chron_ cells exhibit signs of exhaustion, such as impaired cytokine production and expression of inhibitory receptors. We observed an almost complete loss of IFNγ and TNFα cytokine secretion by the fourth activation in T_chron_ as compared to T_re-act_ (Figure 1(b)). Notably, while the viability of T_chron_ cells modestly declined with each activation, the magnitude of cytokine loss was significantly greater than the loss of viability (Supplementary Figure S1a,b). We also observed high expression of the inhibitory receptors LAG3, TIM3, PD1, and CD39 in T_chron_ cells (Figure 1(c)). Of note, T_re-act_ cells also expressed high levels of these markers, which are known to be expressed transiently during T cell activation (Figure 1(c)). Interestingly, there was heterogeneity in expression dynamics amongst the inhibitory receptors in T_chron_ cells, with some oscillating between high and low expression levels with each activation and rest interval (PD1), while the others gradually increased in expression throughout the course of the 2 weeks (LAG3, TIM3, and CD39) (Supplementary Figure S1c).

Exhausted CD8+ T cells possess a transcriptional programme that is distinct from effector, naive, and memory T cells. We therefore performed RNA sequencing (RNA-Seq) on T_chron_, T_re-act_, and T_acute_ cells in four healthy donors to determine if T_chron_ cells exhibited canonical features of exhaustion. Principal component analysis (PCA) revealed that T_acute_ cells are transcriptionally distinct from T_re-act_ and T_chron_ cells (Figure 1(d)). T_re-act_ cells also form a well-defined cluster. Interestingly, in two donors, T_chron_ cells segregated from both T_acute_ and T_re-act_ cells, while in the other two donors they cluster near T_re-act_. We wondered if this meant the *in vitro* exhaustion protocol did not induce chronic activation to the same degree in the latter two donors. CD8+ exhausted T cells are heterogeneous, gradually differentiating from a more stem-like progenitor exhausted state to a terminally exhausted state. Thus, the two donors that cluster near the T_re-act_ cells may represent a different stage along this exhaustion trajectory compared to the other two donors. To explore this hypothesis, we examined gene expression of exhaustion-associated genes at the level of individual donors to illuminate any differences between them. We compared the two donors clustering near T_re-act_ cells to the two donors segregating from both T_re-act_ and T_acute_ cells (Supplementary Figure S2a, dashed and solid lines, respectively). We refer to these donors as T_chron1_ and T_chron2_, respectively (Supplementary Figure S2a). The transcription factor TCF1, encoded by the *TCF7* gene, is the key regulator for maintaining the progenitor-like exhausted T cell population (Z. Chen et al., [20]; Im et al., [21]; Utzschneider et al., [22]; Wu et al., [23]). Interestingly, *TCF7* expression was elevated in T_chron1_ donors relative to T_chron2_ donors under chronic stimulation (Supplementary Figure S2b). In contrast, the mRNAs for transcription factors and transcription regulators TOX2, NR4A2, and ID2 (*TOX2, NR4A2, ID2)*, which have been associated with exhausted and terminally differentiated states, are more highly expressed in T_chron2_ donors (Supplementary Figure S2b) (J. Chen et al., [24]; Knell et al., [25]; Omilusik et al., [26] [27]). Similarly, inhibitory receptors (*LAG3, HAVCR2, TIGIT)* were more highly expressed in T_chron2_ donors in the chronic condition (Supplementary Figure S2b). While the cytokine *TNF* was downregulated in the chronic condition in all donors, the degree of downregulation was significantly greater in T_chron2_ donors (Supplementary Figure S2b). Taken together, these data suggest a more progenitor-like state for the T_chron1_ donors compared to the T_chron2_ donors. Importantly, however, not all exhaustion markers were enriched in T_chron2_ donors. Some were equally expressed in all T_chron_ donors (*PDCD1, CCR7, SOX4)*, while *SELL* and *ID3*, generally associated with less differentiated T cell states, were more highly expressed in T_chron2_ donors (Supplementary Figure S2b) (Gago Da Graça et al., [28]; Tsui et al., [29]). Taken together, these data suggest that chronic activation induced heterogeneous exhaustion-associated transcriptional states in T_chron1_ and T_chron2_ donors.

We also compared all T_chron_ to T_re-act_ donors to identify differentially expressed genes (Figure 1(e)). In line with our observation that cytokine production is severely hindered in T_chron_ cells, both the *IFNG* and *TNF* transcripts were downregulated. We also observed upregulation of mRNAs encoding proteins previously linked to T cell exhaustion, such as the transcription factors TOX2 and SOX4, membrane glycoprotein LAYN, and ectoenzyme CD38 [27,30–33]. Interestingly, the mRNA for transcription factor Eomes (*EOMES*) was downregulated in T_chron_ cells in our data. Although T cell exhaustion is generally associated with elevated expression of Eomes, its expression varies across exhaustion states [34]. Progenitor and intermediate exhausted T cells express lower levels of Eomes, whereas terminally exhausted cells exhibit high Eomes expression [35]. Overall, these data suggest that T_chron_ cells may not adopt a uniformly exhausted state, but nevertheless exhibit many hallmarks of exhaustion.

Finally, we used gene set enrichment analysis (GSEA) to probe whether certain pathways and biological processes were enriched in T_chron_ or T_re-act_ cells. Using the Molecular Signatures Database hallmark human gene set collection, we obtained a broad overview of the transcriptional programmes associated with chronic stimulation (Liberzon et al., [36]) (Supplementary Table S1). We were particularly intrigued by the strong negative enrichment of MYC transcription factor target genes in T_chron_ cells (Figure 1(f)). MYC is a major driver of metabolic reprogramming during T cell activation and is required for cell growth and proliferation (R. Wang et al., [37]). While the role of MYC in T cell exhaustion is not well understood, *MYC* mRNA has been reported to be reduced in exhausted T cells (Wherry et al., [38]). Experimental downregulation of *MYC* is also sufficient to induce exhaustion-associated phenotypes (Liu et al., [39]). We also observed negative enrichment of genes involved in mTORC1 signalling and glycolysis in T_chron_ cells (Figure 1(f)). MYC and mTOR pathways have extensive crosstalk, as they cooperate to promote anabolic and glycolytic metabolic programmes that support effector T cell function (Chi, [40]; R. Wang et al., [37]). Thus, the coordinated depletion of MYC targets, mTORC1 signalling genes, and glycolytic genes is consistent with previous reports that metabolic dysfunction contributes to T cell exhaustion [41,42], (Zheng et al., 2022).

### T_chron_ cells translationally downregulate TOP mRNAs

**Figure 2.**
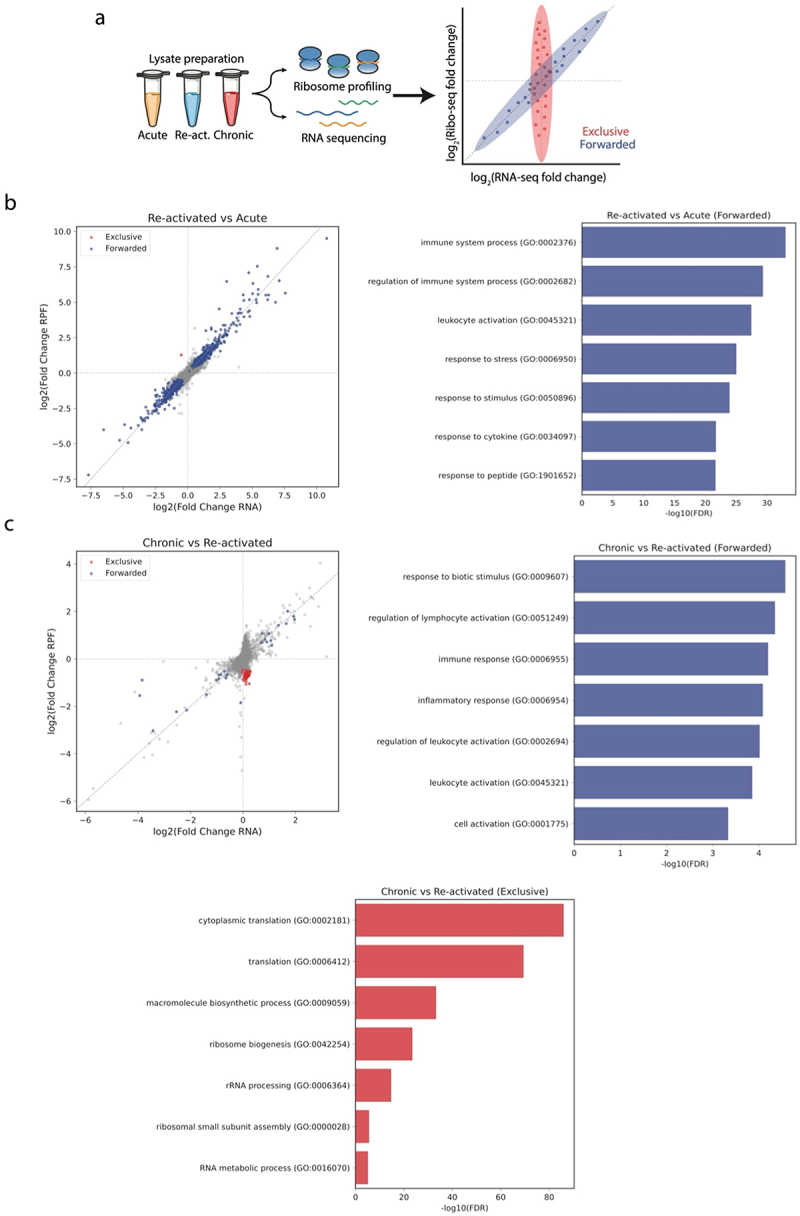
Transcriptional and translational regulation control distinct biological processes in exhausted T cells.

To investigate translation dynamics globally during T cell exhaustion, we also performed ribosome profiling in parallel with RNA-Seq on lysates from T_chron_, T_re-act_, and T_acute_ cells (Figure 2(a)). Ribosome profiling provides a snapshot of genome-wide ribosome occupancy on mRNAs by capturing and deep sequencing the ~30 nucleotide ribosome-protected fragments (RPFs) left after nuclease digestion [43]. The density of RPFs on a transcript is a proxy for the extent of protein synthesis [44]. Here, we used a simplified ribosome profiling workflow that uses P1 nuclease for the digestion step and ordered two-template relay (OTTR) for library preparation [45]. These modifications to the original method greatly reduce the labour and input required to perform ribosome profiling, making it ideal for experiments where cell material is limited [45].

**(a)** Schematic for studying translation regulation in T_acute_, T_re-act_, and T_chron_ cells. Lysates from each condition were prepared from four donors and RNA-Seq and ribosome profiling were performed in parallel. Differential expression analysis using deltaTE reveals genes that are forwarded (blue) and genes that are exclusive (red) [46]. Forwarded genes are regulated primarily at the transcript level, while exclusive genes are regulated primarily at the translational level. **(b)** Scatterplots of log_2_(fold change) RPF counts vs log_2_(fold change) RNA counts, comparing T_re-act_ to T_acute_ (*n* = 5851). Exclusive genes (*n *= 1) indicated in red and forwarded genes (*n* = 772) indicated in blue. Exclusive genes are defined as genes in which ∆mRNA is not significant (FDR > 0.05), ∆RPF is significant (FDR <0.05), and ∆TE is significant (FDR < 0.05). Forwarded genes are defined as genes in which ∆mRNA is significant (FDR < 0.05), ∆RPF is significant (FDR < 0.05), and ∆TE is not significant (FDR > 0.05) [46]. To the right, GO analysis indicates the top biological processes associated with the forwarded genes. **(c)** Scatterplots of log_2_(fold change) RPF counts vs log_2_(fold change) RNA counts, comparing T_chron_ to T_re-act_ (*n* = 5851). Exclusive genes (*n *= 49) indicated in red and forwarded genes (*n* = 26) indicated in blue. GO analyses for the forwarded (blue) and exclusive (red) genes are also shown.

Our ribosome profiling results showed the expected read length distribution for eukaryotic RPFs, averaging around ~35 nt (Supplementary Figure S3a) [45]. The percentage of reads that mapped to mRNA (as opposed to rRNA, gDNA, etc) ranged from ~10–30% (Supplementary Figure S3b). Principal component analysis of RPFs revealed a similar trend to the RNA-seq data – T_acute_ and T_re-act_ cells were distinct populations, while T_chron_ cells showed two donors that clustered with T_re-act_ cells while the other two donors formed a unique cluster (Supplementary Figure S3c and Figure 1(d)).

We then used our ribosome profiling and RNA-seq data to investigate gene regulation during T cell activation and T cell exhaustion programmes (Supplementary Tables 2–4). To this end, we performed an integrative analysis of both our RNA-seq and ribosome profiling datasets using the deltaTE method to compare T_re-act_ to T_acute_ cells and T_chron_ to T_re-act_ cells [46]. Genes were sorted into two regulation classes: forwarded genes, where changes in RPF counts mirrored changes in mRNA counts and therefore indicate transcriptional regulation, and exclusive genes, where changes in RPF counts occur despite no change in mRNA counts and therefore indicate translational regulation [46] (Figure 2(a)). Of note, there are two more regulation classes – buffered and intensified – which we did not observe in our analysis. When comparing T_re-act_ to T_acute_ cells, nearly all the differentially expressed genes (*n* = 773) fell under the forwarded category, suggesting that gene regulation occurs largely at the level of transcription during CD8+ T cell activation (Figure 2(b) and Supplementary Table 4). Consistent with this, gene ontology (GO) analysis revealed that the biological processes enriched among the forwarded genes were those related to immune system regulation and T cell activation (Figure 2(b)). By contrast, comparison of T_chron_ and T_re-act_ cells revealed only 26 forwarded genes, which were again enriched in processes related to immune function (Figure 2(c) and Supplementary Table S4). Interestingly, there were 49 genes categorized as exclusive that showed significant translational repression in T_chron_ relative to T_re-act_ cells (Figure 2(c) and Supplementary Table 4). GO analysis revealed that this group of translationally repressed genes is highly enriched for processes related to translation and ribosome biogenesis, including ribosomal protein genes (*RPS24*, *RPL32*), initiation factor genes (*EIF3E*), and translation elongation factor genes (*EEF1A1*, *EEF1B2*) (Figure 2(c) and Supplementary Table 4).

**Figure 3.**
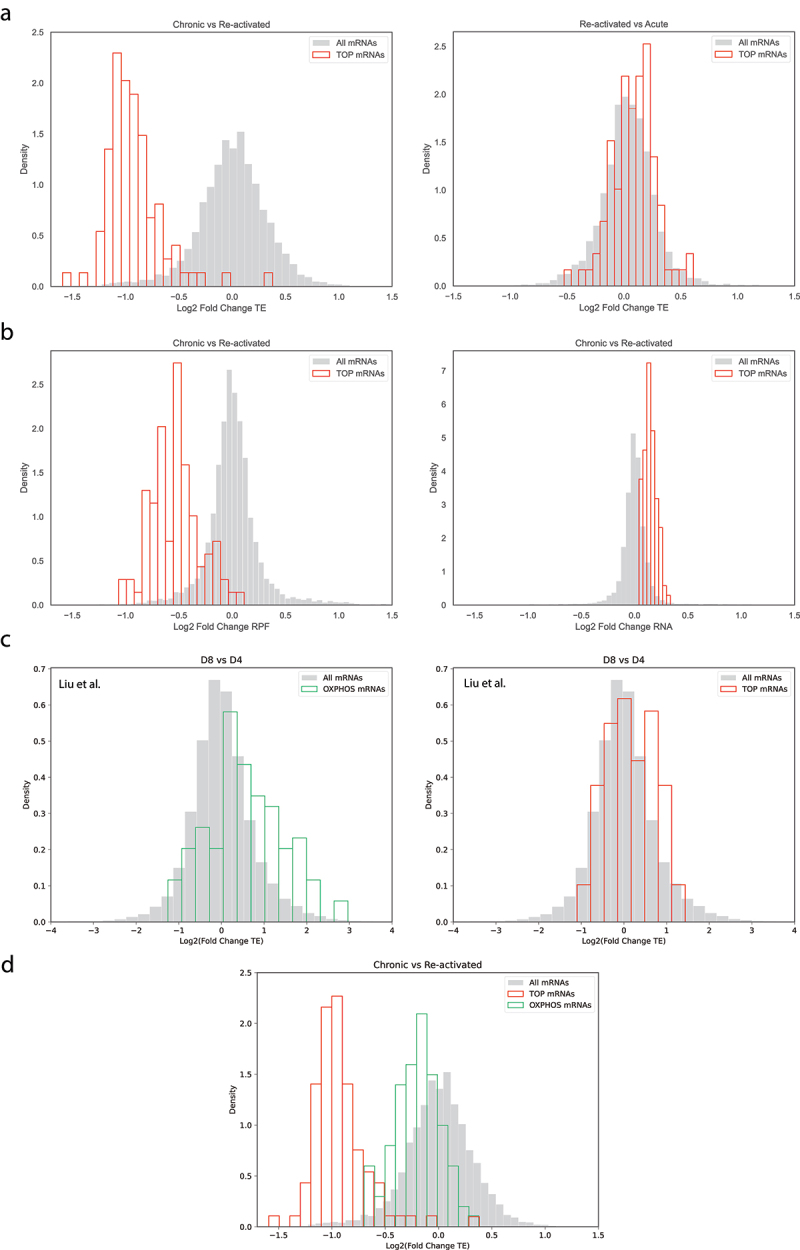
TOP mRNAs are translationally repressed in T_chron_ compared to T_re-act_ cells.

Next, we were interested in further investigating the group of exclusive genes that were translationally downregulated in T_chron_ cells. Importantly, the majority of the genes in this category are mRNAs that contain TOP motifs. We therefore hypothesized that all TOP mRNAs may be subject to dynamic translational control in T_chron_ cells. To test this, we analysed the translation efficiencies (TE) of all genes using deltaTE [46]. TE is defined as the ratio of the number of RPFs mapping to the open reading frame (ORF) over the number of mRNA counts [44,46]. We then compared TEs of T_chron_ to T_re-act_ cells and T_re-act_ to T_acute_ cells (Supplementary Figure S3d, Supplementary Tables S2–3). Strikingly, TOP mRNAs are significantly repressed relative to all other mRNAs when comparing T_chron_ to T_re-act_ cells (Figure 3(a)) [47]. In contrast, TOP mRNAs show no translation regulation during T cell activation (T_re-act_ vs T_acute_) (Figure 3(a)). Notably, the translational repression in T_chron_ occurs despite a mild increase in transcript abundance, indicating that downregulation is driven entirely at the level of translation (Figure 3(b)).

(a) Histograms of log_2_(fold change) TE; all mRNAs in grey (*n* = 5851) and TOP mRNAs (*n* = 94) in red. Comparing T_chron_ to T_re-act_ on the left, comparing T_re-act_ to T_acute_ on the right. (b) Histograms of log_2_(fold change) mRNA and log_2_(fold change) RPF comparing T_chron_ to T_re-act_; all mRNAs in grey (*n* = 5851) and TOP mRNAs (*n* = 94) in red. (c) Histograms of log_2_(fold change) TE values comparing Day 8 (D8) to Day 4 (D4) chronically stimulated CD8+ murine T cells, taken from [15], all mRNAs in grey (*n* = 9382), OXPHOS mRNAs in green (left, *n* = 139), and TOP mRNAs in red (middle, *n* = 94). (d) Histograms of log_2_(fold change) TE comparing T_chron_ to T_re-act_ from this study; all mRNAs in grey (*n* = 5851), OXPHOS mRNAs in green (*n* = 139), and TOP mRNAs in red (*n* = 94).

A recent study by Liu and colleagues used an *in vitro* chronic antigen stimulation model combined with a modified ribosome profiling approach to investigate translational dynamics during T cell exhaustion [15]. We were therefore interested in assessing the similarities and differences observed in their results compared to those presented here. They observed hypertranslation of OXPHOS mRNAs driven by the RNA-binding protein LARP4 in their chronically stimulated CD8+ murine T cells (Figure 3(c), left). However, they did not observe the translational repression of TOP mRNAs we observed (Figure 3(c), right). In contrast to Liu et al., we did not observe the increase in translation efficiency of OXPHOS mRNAs in our T_chron_ cell data. In fact, we observed a mild repression in the translation of these mRNAs (Figure 3(d)). We explore possible reasons for this seeming discrepancy in the Discussion.

### mTOR activity is elevated in T_chron_ cells

**Figure 4.**
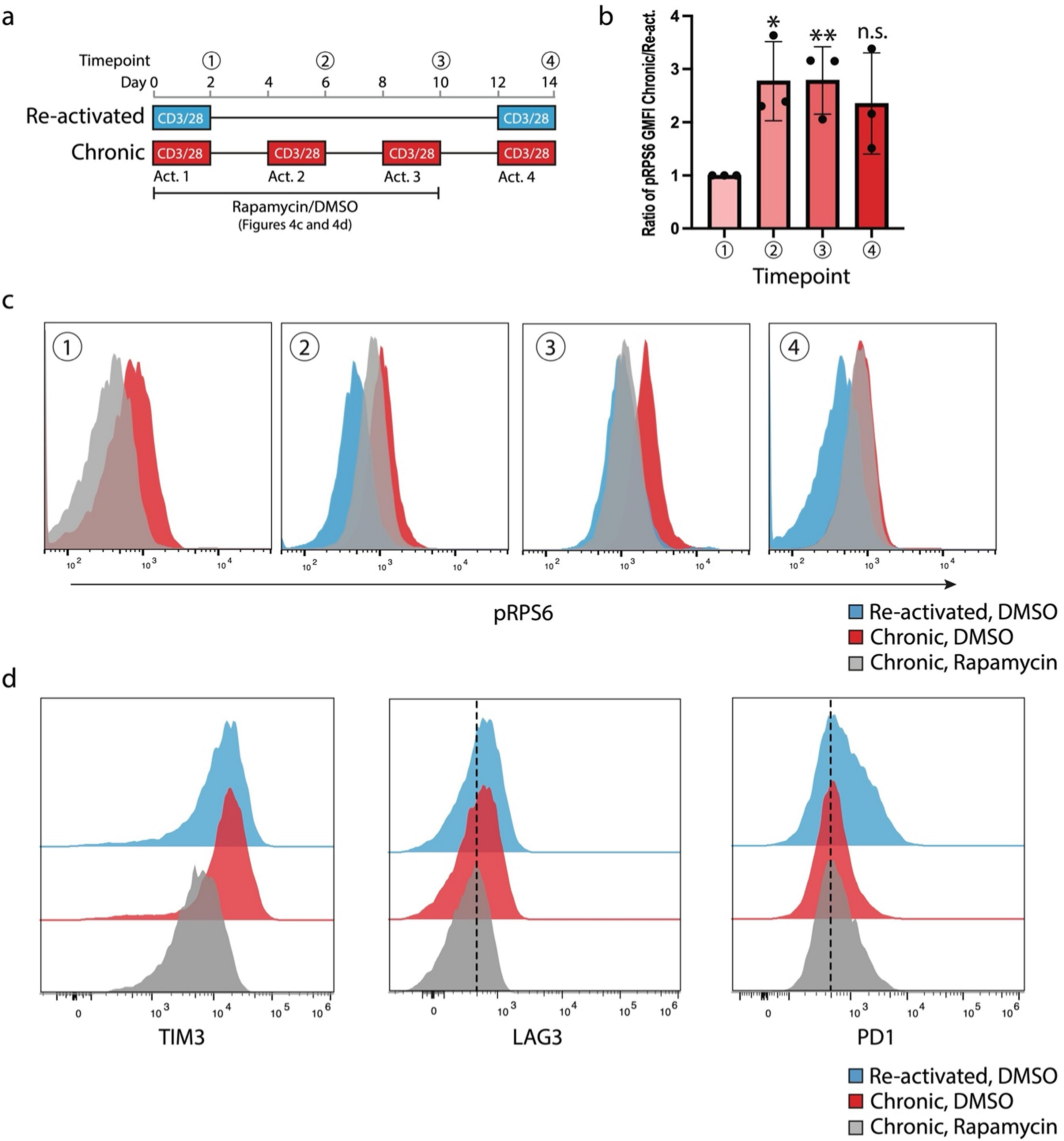
mTOR activity is elevated in T_chron_ compared to T_re-act_ cells.

TOP mRNA translation is highly sensitive to the activity of mTOR. Given our observation that TOP mRNAs are translationally downregulated in T_chron_ cells, we sought to investigate the role of mTOR signalling during this process. To do so, we first assessed the state of mTOR activity using phosphorylation of ribosomal protein S6 (pRPS6) as a readout. We hypothesized that reduced mTOR activity in T_chron_ relative to T_re-act_ cells underlies the translational repression of TOP mRNAs that we observed in our ribosome profiling and RNA-seq data. This would also match the negative enrichment of mTORC1 signalling pathway genes that we observed in T_chron_ cells from GSEA of our RNA-seq data (Figure 1(f)). Unexpectedly, we observed the opposite effect – pRPS6 levels were consistently higher in T_chron_ than in T_re-act_ cells throughout activations 2, 3, and 4 (Figure 4(a,b)). Notably, T_chron_ cells have greater levels of pRPS6 even after the last activation (activation 4), during which T_re-act_ cells were also subjected to the same activation (Figure 4(a,b)).

(a) Schematic for *in vitro* generation of exhausted T cells with timepoints at which flow cytometry measurements were taken, labelled (1–4). Cells were treated with rapamycin (or DMSO) during the first three activations for panels c and d. (b) The ratio of the geometric mean fluorescence intensity (GMFI) of phospho-RPS6 for T_chron_ over T_re-act_ cells at each timepoint (*n *= 3 donors, error bars represent mean ± standard deviation). *p* values were calculated using unpaired two-tailed t tests compared to timepoint 1 (*p < 0.05, **p < 0.01, *n.s*.=not significant). (c) Representative flow cytometry histograms of phospho-RPS6 at each timepoint (*n* = 3 donors). (d) Representative flow cytometry histograms of PD1, LAG3, and TIM3 (*n *= 3 donors). The data shows the last time point (day 14).

Next, we used an inhibitor of mTOR, rapamycin, to test two alternative hypotheses based on our seemingly contradictory observations. First, given our observation that TOP mRNAs are repressed in T_chron_ cells, we hypothesized that inhibiting mTOR activity via rapamycin treatment would push these T cells towards an exhausted state by further repressing translation of TOP mRNAs. Alternatively, given our observation that mTOR activity is constitutively high in T_chron_ cells, we hypothesized that rapamycin treatment would put a brake on mTOR activity and slow down the progression of T cell exhaustion. We limited rapamycin treatment to the first three activations (Figure 4(a)), consistent with studies showing that rapamycin treatment improves outcomes when administered to mice during the initial phase of chronic infection, but has the opposite effect when administered after the establishment of exhaustion [48]. Notably, rapamycin treatment reduced pRPS6 levels compared to the DMSO treated control in T_chron_ cells (Figure 4(c)), consistent with mTOR inhibition. However, after the last activation, at which point rapamycin had been removed from the media, pRPS6 levels in T_chron_ cells returned to the same level as the DMSO treated control T_chron_ cells (Figure 4(c)). We also observed a smaller cell size during rapamycin treatment of T_chron_ cells as measured by forward scatter (FSC), consistent with the role of mTOR in regulating cell growth (Supplementary Figure S4a, timepoint 2). Once again, this effect was transient and disappeared upon rapamycin removal (Supplementary Figure S4a, timepoint 4). Notably, rapamycin treatment improved cell viability throughout the 2-week activation period as compared to T_chron_ cells treated with DMSO, as observed by periodic cell counting. We also used inhibitory receptor expression to assess the effect of rapamycin treatment on T_chron_ cells. Intriguingly, we observed a reduction in LAG3 and TIM3 expression, but not PD1 expression, in the rapamycin treated sample as compared to the DMSO treated control after the final activation (Figure 4(d) and Supplementary Figure S4b). This result better matches our second hypothesis – that rapamycin treatment effectively restrains mTOR activity to slow the progression of T cell exhaustion.

## Discussion

In this study, we performed RNA-sequencing and ribosome profiling in parallel to investigate changes in translation efficiency that occur during T cell exhaustion. Our *in vitro* method for generating exhausted CD8+ T cells effectively recapitulates key hallmarks of T cell exhaustion, including reduced cytokine production, inhibitory receptor expression, and changes in transcriptional programming. Moreover, similar results have been reported by other groups using chronic stimulation to model T cell exhaustion, highlighting the reproducibility and robustness of this method [15–19]. Importantly, this approach is simple and can produce the large number of cells necessary for genome-wide approaches such as ribosome profiling.

Our results reveal a striking downregulation in translation efficiency of genes encoding ribosomal proteins and other translation factors during chronic activation of CD8+ T cells. Intriguingly, the majority of the mRNAs encoded by these genes contain a unique feature at their 5’ end known as the TOP motif. Indeed, our data show that nearly all the canonical TOP-motif-containing mRNAs were translationally repressed in T_chron_ cells compared to T_re-act_ cells. Notably, this repression occurred only at the translational level. In fact, these transcripts were mildly stabilized in the chronic condition as compared to the re-activated condition. Our findings provide a new perspective on the dynamic regulation of TOP mRNAs in T cell biology. TOP mRNAs are some of the most abundant transcripts in naive T cells, and yet their translation is repressed until antigen stimulation, at which point their translation is rapidly induced to support ribosome biogenesis [1,3,14]. As the effector response reaches its peak, TOP mRNA translation is downregulated again as the cells stop proliferating [1]. Our results suggest that a similar repression occurs during chronic T cell stimulation, perhaps indicating an adaptive response to help sustain cell survival under prolonged antigen exposure.

TOP mRNA translation is regulated by the mTOR pathway. Interestingly, we show that mTOR activity is sustained across all four activations in T_chron_ cells, seemingly in opposition to the inhibition of TOP mRNA translation and negative enrichment of mTOR pathway genes that we observed. Studies in mouse models of acute and chronic viral infection have reported highly dynamic mTOR activity throughout the progression of exhaustion. During both acute and chronic infections, mTOR signalling initially peaks at the height of the effector response [41,42]. However, in an acute infection, mTOR signalling rapidly returns to baseline as the infection is cleared, whereas in a chronic infection, mTOR signalling persists – albeit at a lower level – due to ongoing antigen stimulation [41,42]. Notably, during chronic infection, T cells lose the ability to induce new mTOR activity upon restimulation, while T cells responding to an acute infection maintain robust mTOR signalling capabilities [41,42]. Importantly, evidence of elevated mTOR signalling (by phosphorylation of RPS6 and enrichment of mTOR-associated transcriptional programmes) does not necessarily equal mTOR responsiveness [41]. This distinction may help reconcile the seeming discrepancy between sustained mTOR activity in T_chron_ cells and the repression of TOP mRNAs and transcriptional downregulation of metabolic pathway genes that we observed. These findings suggest that mTOR activity becomes uncoupled from its downstream anabolic outputs during chronic stimulation. Consistent with this idea, Bengsch and colleagues observed impaired glucose uptake and glycolysis, despite persistent mTOR signalling [41]. Together, these results suggest a model in which mTOR activity functions like a gas pedal in a car. Chronic TCR stimulation forces the cellular ‘engine’ to run continuously for a long time, eventually leading to fuel shortage and damage to the machinery. At this point, even when the pedal is depressed, the engine is no longer responsive. This idea is also supported by the heterogeneity of exhausted T cell populations, where differences in mTOR activity lead to different functional outcomes. Progenitor exhausted T cells exhibit suppressed mTOR activity in the earlier stages of the T cell response compared to their more terminally exhausted counterparts, which preserves their long-term fitness by maintaining responsiveness to mTOR signalling [49]. It should also be noted that while phosphorylation of RPS6 was used as a proxy for mTOR activity in the current study (and in the other studies cited here), a more comprehensive study of mTOR activity will be necessary to fully evaluate its state in chronically activated or exhausted T cells. For example, other phospho-targets of mTOR include eIF4E-binding protein 1 (4E-BP1) and S6 kinase 1 (S6K1).

We further probed the connection between TOP mRNAs, mTOR, and T cell exhaustion by using rapamycin to inhibit mTOR activity. Our results show that rapamycin treatment slows the progression of exhaustion, as evidenced by a reduction in inhibitory receptor expression. Here, we suggest that rapamycin acts as a ‘brake’ during the early phase of chronic activation to preserve the cellular ‘engine’ from burning out. Similar observations have been reported by other groups. Rapamycin treatment during the early stages of a mouse model for chronic viral infection leads to better outcomes by promoting the formation of stem-like CD8+ T cells [48,49]. In contrast, inhibiting mTOR activity later in the infection, when exhaustion was already established, repressed immunity by decreasing the numbers of effector-like T cells [48,49]. Further work will be necessary to determine how mTOR inhibition affects TOP mRNA regulation during the exhaustion program. It will also be informative to test whether the effects observed are recapitulated by Torin1, an ATP-competitive inhibitor that more completely inhibits mTOR activity (Thoreen et al., [50]).

The mechanisms responsible for TOP mRNA repression during chronic T cell activation require further investigation. One candidate is La-related protein 1 (LARP1), an RNA-binding protein that has been identified as the mTOR-sensitive repressor of TOP mRNA translation [51,52]. LARP1 also stabilizes these transcripts in a highly selective manner [53–55]. Under conditions of mTOR inhibition, LARP1 binds the 5’ m^7^G cap and adjacent TOP motif of TOP mRNAs, blocking assembly of the eIF4F complex and thereby inhibiting translation initiation [51,56,57]. Initial models suggested that mTOR regulates LARP1’s binding activity by phosphorylating it at multiple residues and causing its disassociation from TOP mRNAs [58,59]. However, more recently it was found that LARP1 acts as a ribosome sensor by directly binding non-translating ribosomes [60, Saba et al., 2026]. Intriguingly, ribosome binding is necessary and sufficient *in vitro* for TOP mRNA binding, repression, and stabilization by LARP1 (Saba et al., [61]). Saba et al. showed that TOP-LARP1-ribosome complexes form during any condition which leads to greater numbers of free, idling ribosomes. This could occur even under conditions of normal mTOR activity, for example, by knocking down eIF4E to inhibit translation initiation [60]. These findings raise the possibility that LARP1 may be capable of inhibiting TOP mRNA translation during T cell exhaustion even under conditions of high mTOR activity by sensing the presence of free ribosomes. This hypothesis is particularly intriguing in light of evidence from Wolf et al. [3] that naive T cells contain both large numbers of idling ribosomes and repressed mRNAs, the majority of which are regulated by mTOR. Future work will be necessary to determine whether LARP1 contributes to TOP mRNA repression during T cell exhaustion and whether this activity is driven by the accumulation of free ribosomes.

Another interesting avenue for exploration is the connection between MYC transcription factor and translation regulation during chronic T cell stimulation. We observed strong downregulation of MYC target genes in T_chron_ cells in our RNA-seq data (Figure 1(f)). MYC coordinates ribosome biogenesis and translation by regulating transcription of rDNA, ribosomal protein, translation initiation factor, and elongation factor genes (Van Riggelen et al., [62]). MYC also controls the expression of amino acid transporters that are critical for sustaining growth and proteome remodelling during T cell activation (Marchingo et al., [63]). The loss of these MYC-driven processes suggests that multiple pathways, including MYC and mTOR signalling, converge during T cell exhaustion to counteract persistent TCR activation programming.

We were also interested in comparing our ribosome profiling results to those published recently by Liu et al. [15]. Their findings contrast with ours, as they reported hypertranslation of OXPHOS and cytoplasmic translation genes during chronic activation. Interestingly, both Liu et al. and Wang et al. [16] also reported elevated global protein synthesis rates during chronic stimulation. By contrast, we observed a mild repression of OXPHOS mRNAs and a marked repression of TOP mRNAs. These discrepancies may reflect differences in experimental design. Liu et al. chronically stimulated murine CD8+ T cells with anti-CD3 antibody (following an initial 48h anti-CD3/CD28 activation) continuously for 6 days without rest, whereas our study used human CD8+ T cells activated with both anti-CD3 and anti-CD28 antibodies for 14 days with intermittent rest periods. The inclusion of CD28 costimulation represents an important distinction, as CD28 activates PI3K-AKT-mTOR signalling and promotes metabolic programmes that support T cell growth and function (Frauwirth et al., [64]). Consequently, chronic stimulation in the presence or absence of CD28 costimulation may produce distinct translational states, complicating direct comparisons between the two studies. Nonetheless, the stark contrast is intriguing and may reflect underlying biological differences. A possible explanation is that Liu et al. captured an earlier stage of the exhaustion trajectory that precedes the state observed here. Under this hypothesis, chronic stimulation initially promotes a global rise in translational activity and increased synthesis of translational machinery components, leading to excess ribosome accumulation. Over time, the large number of idling ribosomes is sensed by LARP1, which causes formation of repressive TOP-LARP1-ribosome complexes and consequent translational inhibition of TOP mRNAs. This model remains untested and will require further exploration. Future studies combining ribosome profiling with direct measurements of global protein synthesis across a more comprehensive time course, from the onset of exhaustion until the late stages, will be important for determining whether an initial phase of hypertranslation is followed by a state of translational repression.

**Figure 5.**
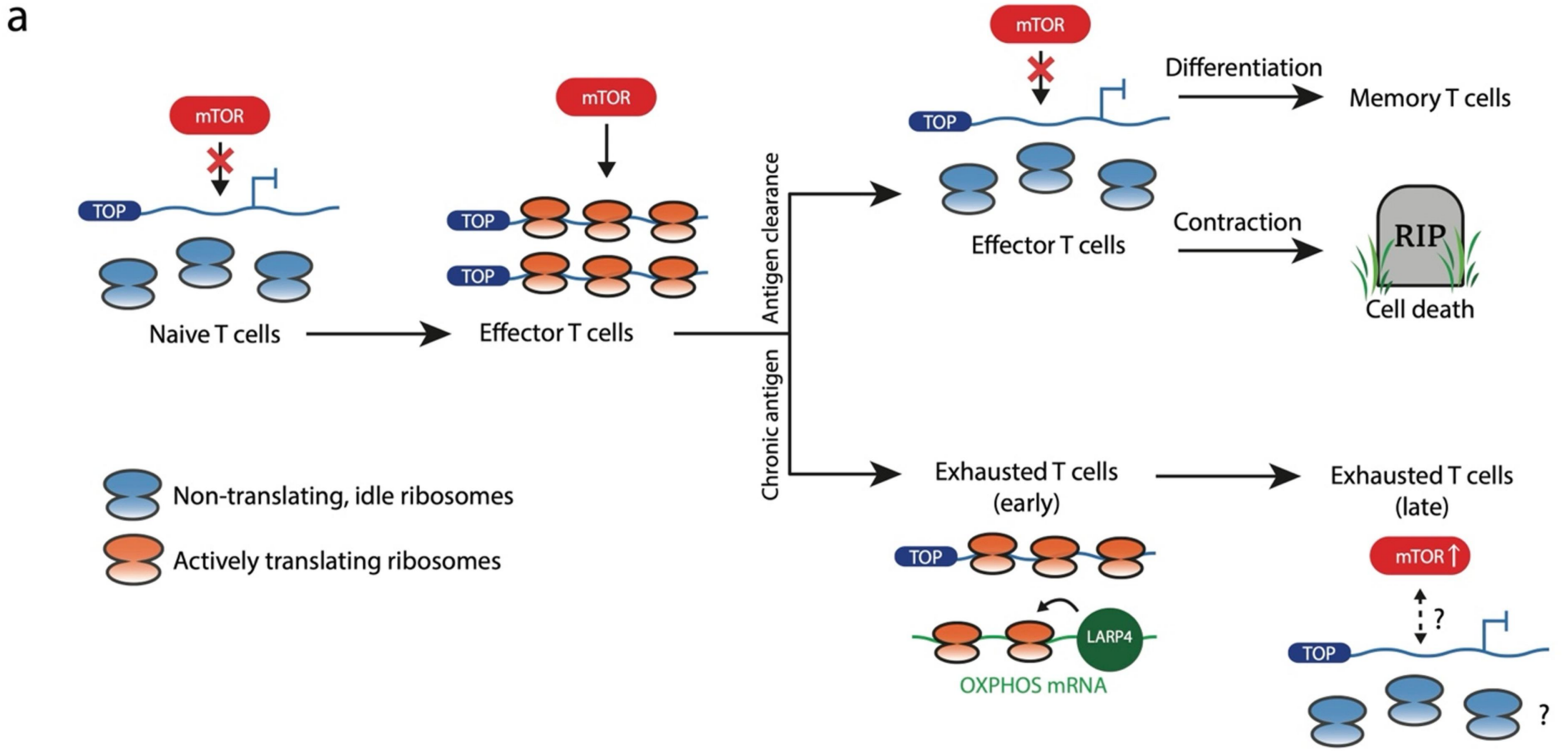
Model of translation regulation during T cell exhaustion.

Finally, we synthesize the findings of several groups, as well as those presented here, to present a model for the role of translation regulation of TOP mRNAs during CD8+ T cell differentiation and exhaustion (Figure 5(a)). TOP mRNAs are abundant but repressed in naive T cells [3,14]. Naive T cells also contain large numbers of idle ribosomes, a strategy that enables a rapid transition to a translationally active state upon TCR stimulation [3]. Following antigen stimulation, TOP mRNAs are rapidly recruited to polysomes and translated in an mTOR-dependent manner as the cells clonally expand and differentiate into effector T cells [1,3]. Araki and colleagues demonstrated that CD8+ T cells downregulate TOP mRNAs at the peak of the effector response following antigen clearance [1]. Notably, even among CD8+ effector T cells, terminal effectors – those that will eventually undergo apoptosis during the contraction phase – and memory precursors – those that will differentiate into long-lived memory T cells – exhibited distinct translational regulation of TOP mRNAs [1]. In contrast, during chronic antigen exposure, TOP mRNAs remain highly translated [1,15,16]. We hypothesize that sustained hypertranslation at this stage eventually triggers a feedback mechanism leading to TOP mRNA repression during the later stages of exhaustion, even in the presence of high mTOR activity.

(a) Schematic of TOP mRNA regulation during the stages of T cell differentiation. Naive T cells contain large numbers of free ribosomes, and TOP mRNAs are repressed [3]. Upon antigen stimulation, TOP mRNAs are rapidly engaged by ribosomes and translated within polysomes [1,3,14]. During an acute infection, TOP mRNAs are repressed following antigen clearance, coinciding with the peak of the effector response – a process sensitive to mTOR activity [1]. During the earlier stages of a chronic infection, TOP mRNAs and OXPHOS mRNAs are highly translated [1,3,15]. The hypertranslation of OXPHOS mRNAs is mediated by the RNA-binding protein LARP4 [15]. In contrast, at later stages, TOP mRNAs are repressed despite high mTOR activity.

## Methods

### Cell culture

Human primary T cells were maintained in ImmunoCult™-XF T cell expansion medium (STEMCELL Technologies). Recombinant human IL-2 (Peprotech) was added to the media at the indicated concentrations. Cells were maintained between 2 × 10^5^ − 1 × 10^6^ cells/mL.

### Isolation of human primary T cells

Peripheral blood mononuclear cells (PBMCs) were isolated from human peripheral blood leukopaks (STEMCELL Technologies). Briefly, the contents of the leukopak were combined with an equal volume of MACS buffer (PBS, 2% FBS, 1 mM EDTA) and spun down (15 min, 100 × g, Dec = 1), following which the pellet was washed 1–2 times (5 min, 400 × g) in the MACS buffer. Bulk (CD3^+^) T cells or CD8^+^ T cells were isolated from the PBMCs by magnetic negative selection using the EasySep™ Human T cell isolation kit or Human CD8^+^ T cell isolation kit (STEMCELL Technologies), respectively, following the manufacturer’s protocol. For future use, cells were frozen in CryoStor® freezing medium (BioLife Solutions) and treated in the same manner as freshly isolated cells once thawed.

### *In vitro* chronic activation of CD8+ T cells

To model T cell exhaustion *in vitro*, CD8^+^ T cells were cultured under repeated stimulation conditions, as shown in Figure 1(a). Cells were stimulated using plate-bound anti-CD3 (clone UCHT1) (Tonbo Biosciences (now Cytek)) at 5 µg/mL and soluble anti-CD28 (clone CD28.2) (Tonbo Biosciences (now Cytek)) at 2.5 μg/mL antibodies for 48 h in ImmunoCult™ T cell expansion medium (STEMCELL Technologies) supplemented with recombinant human IL-2 (200 U/mL). After stimulation, cells were centrifuged and resuspended in fresh medium containing IL-2 (200 U/mL) and expanded for 48 h. This stimulation and rest cycle was repeated four times over the course of 2 weeks. Cell lysates were collected 48 h after the final stimulation. This condition is referred to as the ‘chronic’ stimulation group and models T cell exhaustion.

In parallel, two additional conditions were maintained. The ‘acute’ condition received only a single 48-h stimulation at the beginning of the culture period, followed by expansion in IL-2-containing medium without further stimulation. The ‘re-activated’ condition received an initial stimulation followed by expansion, and then a final stimulation at the end of the 2-week period. All cultures were maintained under identical IL-2 concentrations (200 U/mL) and medium conditions throughout.

### Rapamycin treatment

Rapamycin was added to the media during both the stimulation and rest stages at 10 nM during the first three activations in T_chron_. As a control, we included a condition in which an equal volume of DMSO as in the rapamycin treatment was added to the media during the first three activations.

### Enzyme-linked immunosorbent assay (ELISA)

We used ELISAs to quantify cytokine production (Interferon gamma (IFN-γ) and Tumor Necrosis Factor alpha (TNF-α)) by T_chron_ and T_re-act_ cells following each stimulation. At each stimulation, cells were counted and plated in triplicate at 2 × 10^5^ cells/well in a 96-well flat bottom plate coated with anti-CD3 antibody (5 µg/mL). Anti-CD28 antibody (2.5 µg/mL) and IL2 (200 U/mL) were added directly to the media containing the cells. After 48 h, the cell supernatants from these wells were collected and stored in the −80°C freezer until use. Cell supernatants were diluted appropriately and the ELISA MAX™ Human IFN-γ and TNF-α Sets (BioLegend) were used to determine IFN-γ and TNF-α concentrations following the manufacturer’s protocol. Absorption at 450 nm was measured using a TECAN Spark plate reader. The IFN-γ and TNF-α standards provided in the kits were used to generate a standard curve. GraphPad Prism was used to fit the standard curve.

### Flow cytometry

Flow cytometric analysis was performed on an Attune NxT Acoustic Focusing Cytometer (ThermoFisher). Briefly, cells were pelleted in a 96-well round bottom plate and washed with PBS before staining. For intracellular staining, cells were fixed and permeabilized using the Cytofix/Cytoperm Fixation/Permeabilization kit (BD Biosciences) according to the manufacturer’s instructions. Cells were stained with the following antibodies and live/dead stain diluted in PBS: LIVE/DEAD fixable near-IR (Invitrogen), CD8 (BioLegend, clone SK1), PD-1 (BioLegend, clone EH12.2H7), TIM3 (BioLegend, clone A18087E), LAG3 (BioLegend, clone 11C3C65), CD39 (BioLegend, clone A1), and RPS6 phospho Ser235/236 (BioLegend, clone A17020B). Cells were stained for surface markers on ice for 20 min in the dark. Cells were washed twice in PBS and resuspended in 150 µl of PBS for analysis. Flow cytometry data was analysed using FlowJo software.

### RNA-seq

Total RNA was extracted from the same lysates collected for ribosome profiling using the Direct-zol RNA miniprep kit (Zymo Research). Library preparation and sequencing were carried out by Novogene using an Illumina NovaSeq X Plus 150PE platform. The FASTQ files provided by Novogene were already trimmed and filtered. Reads were aligned to a custom transcriptome that includes both the NCBI RefSeq MANE v0.95 transcript set and the 13 mitochondrial mRNA sequences from Ensembl [45]. We used Salmon to quantify transcript-level abundances [65]. PyDESeq2 was used for differential expression analysis [66]. Gene set enrichment analysis (GSEA) was performed using GSEA software, with the hallmark human gene sets from the Molecular Signatures Database (MSigDB) (Hallmark Myc Targets V2, Hallmark mTORC1 signalling, Hallmark Glycolysis) (Liberzon et al., 2015; Mootha et al., [67]; Subramanian et al., [68]).

### Ribosome profiling

Samples were prepared according to the protocol described in Ferguson et al. and on the Karnateq website [45]. Briefly, on the day of collection, T cells were pelleted by centrifugation and washed once in PBS. The pellet was then resuspended thoroughly in 1 mL of lysis buffer (20 mM Tris-HCl, pH 7.4, 150 mM NaCl, 5 mM MgCl2, 1 mM DTT, 100 μg/mL cycloheximide, 1% v/v Triton X-100, 25 U/mL Turbo DNase I (Invitrogen)) and digested on ice for 30 min. The cell lysate was triturated through a 26 G needle 10 times and clarified by centrifugation (10 min, 4°C, 20,000 × g). Lysates were aliquoted, snap-frozen in liquid nitrogen, and stored at −80°C until ready for use.

RNA concentration of the lysates was determined using the QuantIt RiboGreen RNA kit (Invitrogen). Lysates were subsequently digested by P1 nuclease (New England Biolabs) at 20 U per µg of RNA for 1 h at 37°C on a tube rotator. Ribosomes were pelleted by sucrose cushion in a TLA 110 rotor at 4°C for 1 h at 100,000 rpm. Trizol was added to the ribosome pellets and RNA was extracted using a modified method called mirRICH to preferentially enrich for small RNA fragments [45,69]. Library generation was carried out using the OTTR cDNA library construction kit (Karnateq and a generous gift from the Collins lab) following the manufacturer’s protocol. 40 ng of RNA per sample was used as input for the OTTR reaction. In parallel, 30 and 40 nucleotide control RNA oligos (a gift from the Collins lab) were used as input for the OTTR reaction to use as size selection markers [45]. cDNA cleanup was performed following RNase A and RNase H digestion using an Oligo Clean & Concentrator kit (Zymo Research). cDNAs were size selected on a 0.6x TBE 8% Urea-PAGE and visualized using the fluorescence of the IR800 dye included in the 5’ adapter. The region between the 30 and 40 nucleotide controls was excised and the cDNA was eluted. Following the protocol described in McGlincy et al., cDNA concentrations were measured by qPCR to determine the number of PCR cycles necessary to amplify each library using Illumina primers [70]. Libraries were quantified using the NEBNext Library Quant Kit, pooled at equimolar concentration, and sequenced by Novogene on a NovaSeq X Plus 150PE platform.

Ribosome profiling computational analysis was carried out as described by Ferguson et al., with minor modifications [45]. First, the adapters were trimmed, unique molecular identifiers (UMIs) were annotated, and reads shorter than 20 nucleotides or of poor quality were discarded using Cutadapt: -m 2 -a AGATCGGAAGAGCACACGTCTGAACTCCAGTCAC -A AGATCGGAAGAGCGTCGTGTAGGGAAAGAGTGT -u 7 -U −7 –rename = ’{id}_NTA = {r1.cut_prefix}’ -m 20 -q 10.

Of note, although the libraries were sequenced with paired-end sequencing, we only used Read 1 for our analysis after this point. Reads were sequentially mapped to rRNA, ncRNA, mtDNA, mRNA, gDNA, size-selection oligos, and OTTR adapter references using Bowtie2. For mRNA, reads were aligned to a custom transcriptome that includes both the NCBI RefSeq MANE v0.95 transcript set and the 13 mitochondrial mRNA sequences from Ensembl [45]. We proceeded with further analysis using only the reads that mapped to mRNA. PCR duplicates were collapsed using UMI-tools [71]. Reads mapping outside of the coding sequence (+15^th^ to −10^th^ codon) were removed using Bedtools intersect [72]. The remaining reads were counted using Salmon [65]. Differential translation efficiency analysis was carried out using DeltaTE [46].

## Supplementary Material

Pledger_Supplementary_Table_3.xlsx

Pledger_Supplementary_Table_1.xlsx

Pledger_Supplementary_Table_2.xlsx

Pledger_etal_SI_Revised_hires.pdf

Pledger_Supplementary_Table_4.xlsx

## Data Availability

The RNA-Seq and ribosome profiling datasets from this work have been deposited in NCBI’s Gene Expression Omnibus (https://www.ncbi.nlm.nih.gov/geo/), entries GSE313691 and GSE313692, respectively.
