# Supplementary material for: Translational repression of 5’ TOP mRNAs in chronically activated CD8+ T cells in vitro
Source: RNA Biol. 2026 Aug 6;23(1):1–18. doi: 10.1080/15476286.2026.2715355 (PMC13479822; doi:10.1080/15476286.2026.2715355)
Supplement: Supplementary file 1 [file KRNB_A_2715355_SM5830.pdf]

Translational Repression of 5' TOP mRNAs in Chronically Activated CD8+ T cells *in vitro*

Erica S. Pledger[1,2], Navya Ambavaram[1,2], Lucas Ferguson[1], Jamie H. D. Cate[1,2,3,4]

[1]Department of Molecular and Cell Biology, University of California Berkeley, Berkeley, CA, USA
[2]Innovative Genomics Institute, University of California Berkeley, Berkeley, CA, USA
[3]Department of Chemistry, University of California Berkeley, Berkeley, CA, USA
[4]Molecular Biophysics and Integrated Bioimaging, Lawrence Berkeley National Laboratory, Berkeley, CA, USA

*To whom correspondence should be addressed: Jamie H. D. Cate (j-h-doudna-cate@berkeley.edu)

Keywords: T cell exhaustion, TOP mRNAs, translation regulation, LARP1, mTOR, ribosome profiling

Supplementary Tables and Figures

Supplementary Table 1. Gene sets used for Gene Set Enrichment Analysis (GSEA). Tabs include MYC target genes, mTORC1 signaling genes, and glycolysis genes. All gene sets came from MSigDB's Hallmark Human Gene Sets (Liberzon et al., 2015).

Supplementary Table 2. Differential translation efficiency of chronic vs. reactivated T cells. Tabs include analysis of ribosome protected fragments (deltaRPF), transcription profiling (deltaRNA), and translation efficiency (deltaTE).

Supplementary Table 3. Differential translation efficiency of reactivated vs. acutely activated T cells. Tabs include analysis of ribosome protected fragments (deltaRPF), transcription profiling (deltaRNA), and translation efficiency (deltaTE).

Supplementary Table 4. Categories of transcriptional and translational changes in activated T cells. Tabs for chronic vs. reactivated T cells and reactivated vs. acute T cells are included.

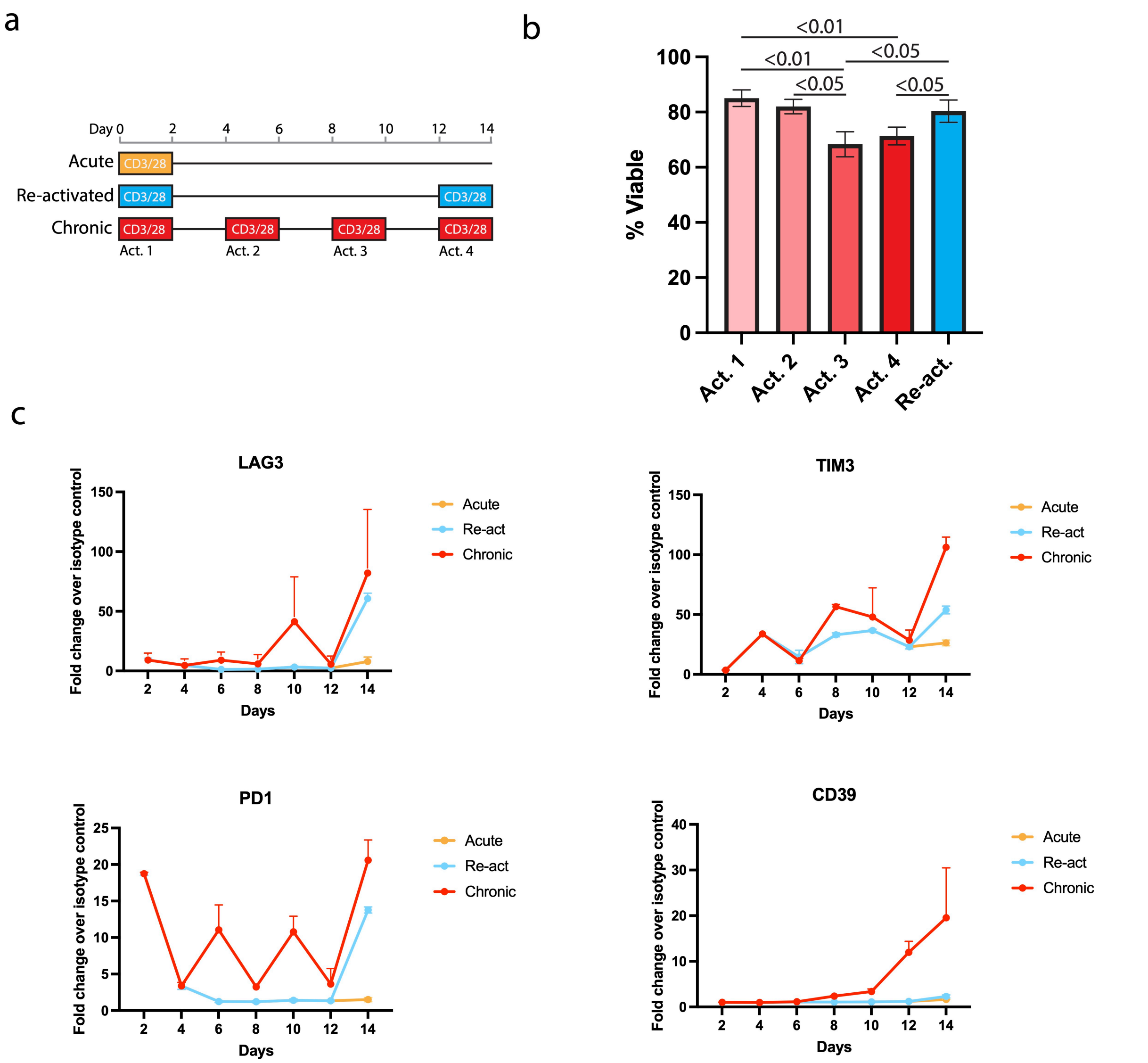

Supplementary Figure 1. ***In vitro* chronically activated CD8+ T cells show hallmarks of T cell exhaustion. (a)** Schematic of T cell activation protocol. **(b)** Viabilities of T_{chron} and $T_{re\text{-}act}$ cells at the end of each 2-day activation. P values were calculated using unpaired two-tailed t tests. **(c)** LAG3, TIM3, PD1, and CD39 expression measured every two days by flow cytometry.

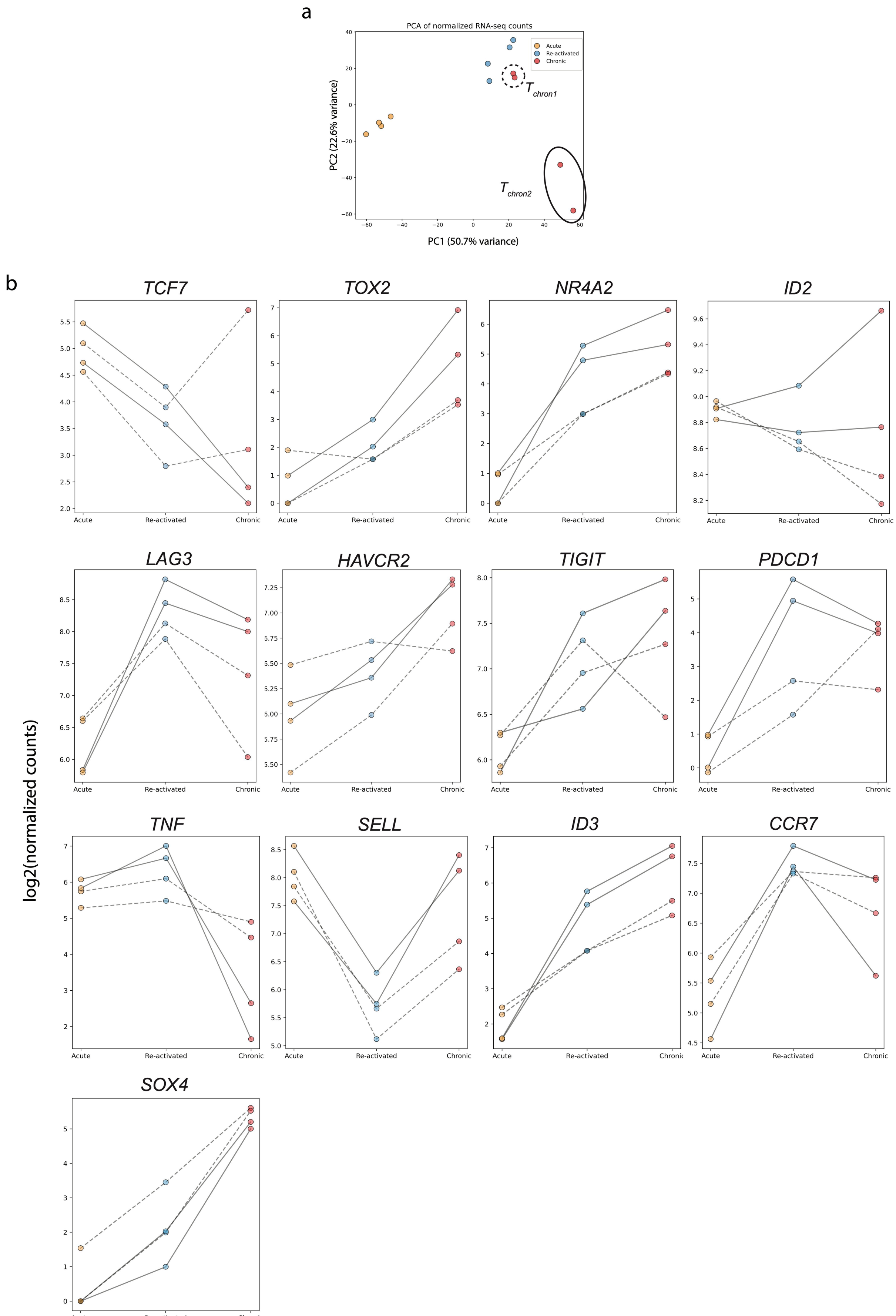
a
PCA of normalized RNA-seq counts
Acute
Re-activated
Chronic
T chron1
T chron2
PC1 (50.7% variance)
PC2 (22.6% variance)
b
TCF7
TOX2
NR4A2
ID2
LAG3
HAVCR2
TIGIT
PDCD1
TNF
SELL
ID3
CCR7
SOX4
log2(normalized counts)
Acute
Re-activated
Chronic

Supplementary Figure 2. Comparison of T_{chron1} and T_{chron2} donors. (a) Comparing the two T_{chron} donors clustering near $T_{re\text{-}act}$ (dashed line, T_{chron1}) to the two T_{chron} donors clustering away from $T_{re\text{-}act}$ and T_{acute} (solid line, T_{chron2}) in PCA. **(b)** log_2(normalized counts) of indicated genes for each donor. Dashed lines represent T_{chron1}. Solid lines represent T_{chron2}.

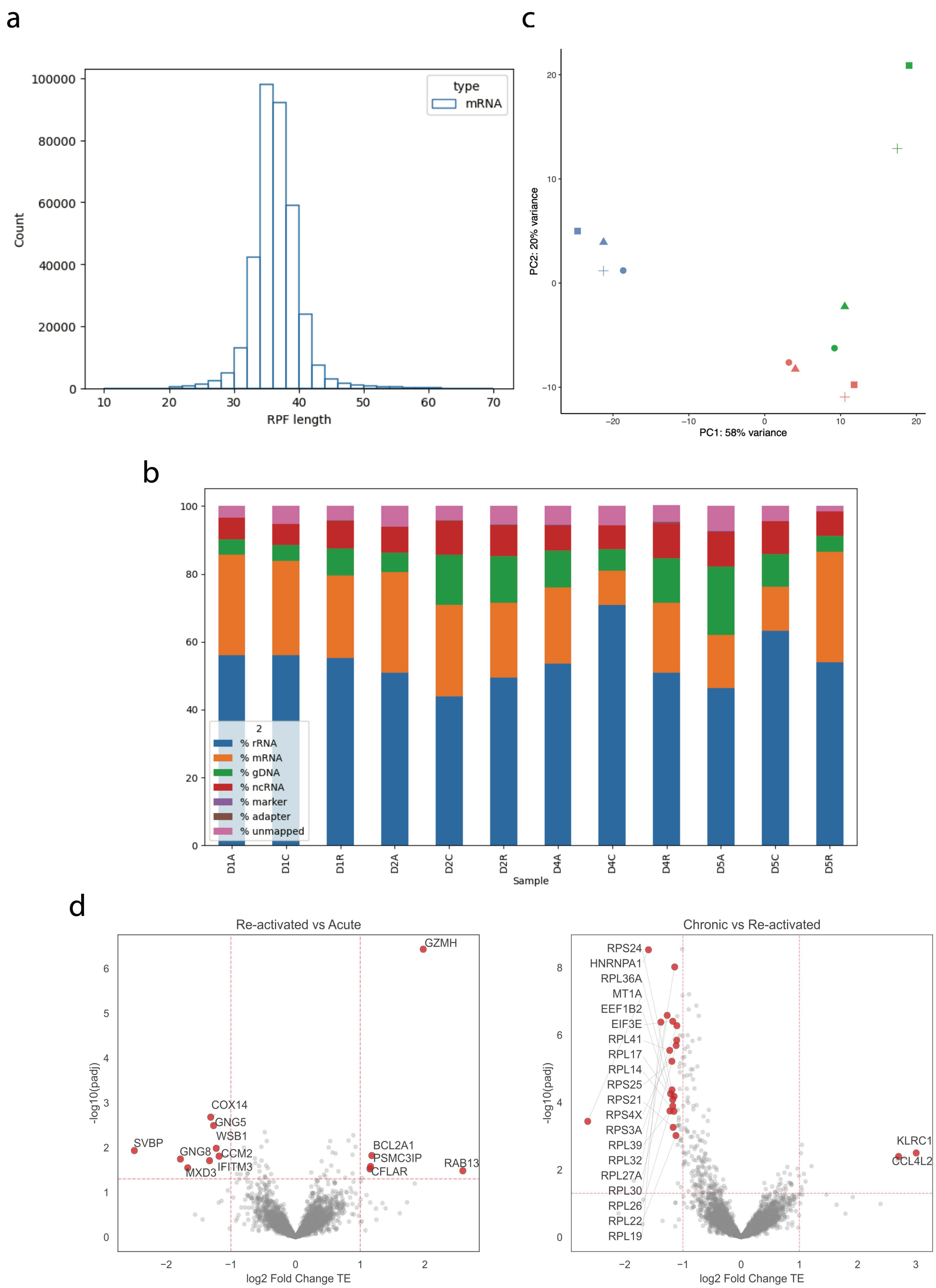

Supplementary Figure 3. Transcription profiling and ribosome profiling statistics and comparisons. (a) Read length distribution of RPFs mapping to mRNA. Data represents only one donor and condition (donor 1, acute); the other donors and conditions showed similar distributions. **(b)** Percentage of RPFs mapping to various

transcript types (rRNA, mRNA, gDNA, etc). **(c)** Principal component analysis of RPFs from T_{acute}, $T_{re\text{-}act}$, and T_{chron} cells in four donors. **(d)** Volcano plots of log_2(fold change) TE comparing $T_{re\text{-}act}$ to T_{acute} (left) and T_{chron} to $T_{re\text{-}act}$ (right).

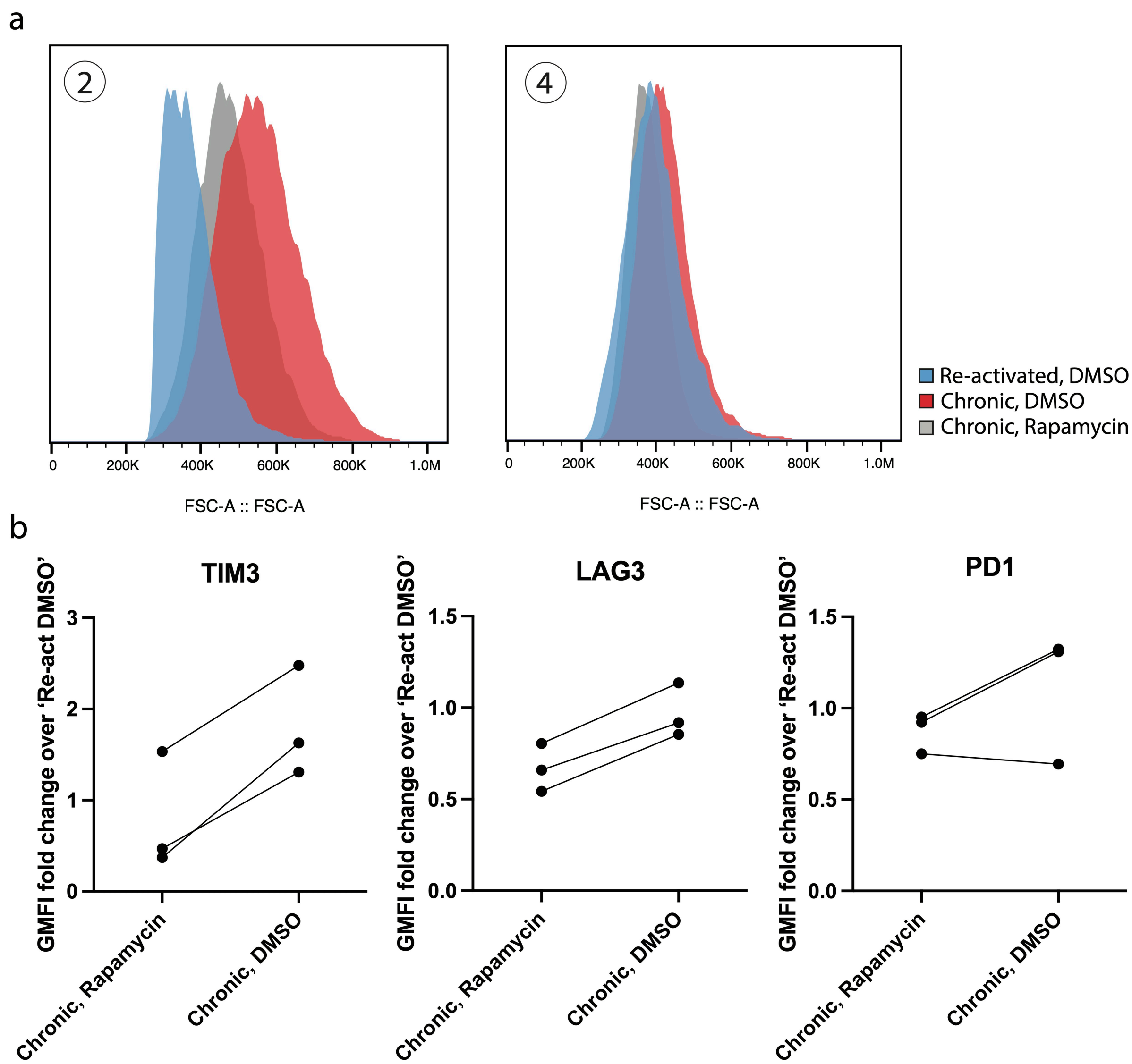

Supplementary Figure 4. Rapamycin treatment slows the progression of T cell exhaustion. (a) Representative flow cytometry histograms of forward scatter (FSC) (n = 3 donors) at timepoints 2 (left) and 4 (right) (timepoints refer to those indicated in Figure 4a). **(b)** Fold change GMFI of TIM3, LAG3, and PD1 expression in T_{chron} cells over $T_{re\text{-}act}$ cells treated with DMSO (n = 3 donors). The data shows the last timepoint (day 14).
